# Viscosity Measurement Sensor: A Prototype for a Novel Medical Diagnostic Method Based on Quartz Crystal Resonator

**DOI:** 10.3390/s21082743

**Published:** 2021-04-13

**Authors:** Andrés Miranda-Martínez, Marco Xavier Rivera-González, Michael Zeinoun, Luis Armando Carvajal-Ahumada, José Javier Serrano-Olmedo

**Affiliations:** 1 Center for Biomedical Technology (CTB), Universidad Politécnica de Madrid (UPM), 28040 Madrid, Spain; marco.rivera@ctb.upm.es (M.X.R.-G.); michael.zeinoun@ctb.upm.es (M.Z.); josejavier.serrano@ctb.upm.es (J.J.S.-O.); 2 Bogotá Campus, Universidad Manuela Beltrán, 110231 Bogotá, Colombia; luis.carvajal@docentes.umb.edu.co; 3 Networking Research Center of Bioengineering, Biomaterials and Nanomedicine (CIBER-BBN), Universidad Politécnica de Madrid (UPM), 28040 Madrid, Spain

**Keywords:** quartz crystal resonator, viscosity, synovial fluid, cerebrospinal fluid, diagnostic sensor

## Abstract

Viscosity variation in human fluids, such as Synovial Fluid (SF) or Cerebrospinal Fluid (CSF), can be used as a diagnostic factor; however, the sample volume obtained for analysis is usually small, making it difficult to measure its viscosity. On the other hand, Quartz Crystal Resonators (QCR) have been used widely in sensing applications due to their accuracy, cost, and size. This work provides the design and validation of a new viscosity measurement system based on quartz crystal resonators for low volume fluids, leading to the development of a sensor called “ViSQCT” as a prototype for a new medical diagnostic tool. The proposed method is based on measuring the resonance frequency at the crystal’s maximum conductance point through a frequency sweep, where crystals with 10 MHz fundamental resonance frequency were used. For validation purposes, artificial fluids were developed to simulate SFs and CFs in healthy and pathological conditions as experiment phantoms. A commercial QCR based system was also used for validation since its methodology differs from ours. A conventional rotational viscometer was used as a reference for calibration purposes. ViSQCT demonstrates the capability to measure the sample’s viscosity differentiation between healthy and pathological fluid phantoms and shows that it can be used as a basis for a diagnostic method of several pathologies related to the studied biological fluids. However, some performance differences between both QCR-based systems compared to the reference system deserves further investigation.

## 1. Introduction

The analysis of the physical properties of human fluids is an ally in the diagnosis of pathologies. In Synovial Fluid (SF) and Cerebrospinal Fluid (CSF), the viscosity change is associated with specific pathologies [1,2,3]. However, the scarce amount of sample obtained is a problem for an accurate and objective measure of the viscosity. The amount of the SF fluid in a joint is no more than 3.5 mL [2,4], and the mean volume of CSF in adults is 150 mL [5]. Since the amount of available biological samples is usually scarce, common viscosity measurement techniques include capillary viscometers or manual techniques like the mucin clot test, or the string test of just forming strips with the syringe (or fingers) tips for the SF viscosity [6]. These techniques still require a large sample volume and/or are not accurate enough, and they need high expertise to follow the procedure and interpret the results correctly. In that sense, a new measurement device is needed, detect viscosity changes in biological fluids, that enhance opportune diagnosis.

Rheumatic diseases as Rheumatoid Arthritis (RA) and Osteoarthritis (OA) represent a problem for the quality of life of the population, causing pain and disability [7]. The macroscopic analysis evaluates the color, transparency, and viscosity of the fluid. At present, the viscosity test involves the extraction of SF through a puncture in the joint using a 21 gauge needle, which is then removed, and the SF is expressed into a test tube. Healthy SF will form a “string” approximately 5 cm long before breaking. SF with poor viscosity will create shorter stings (less than 3 cm) [4,6,8]. This method is a subjective evaluation of viscosity and depends on the laboratory operator’s skills and expertise and the surface conditions where the sample is deposited. SF viscosity depends on Hyaluronic Acid’s (HA) concentration, so at lower concentrations, the viscosity decreases. Some studies relate the SF’s low viscosity with rheumatic diseases, such as as RA and OA [1,2,9].

Meningitis causes an increase in CSF viscosity; therefore, a proper viscosity measurement can help differentiate between healthy CSF, Bacterial Meningitis (BM), and Viral Meningitis (VM) [3]. To diagnose both types of meningitis, it is necessary to perform a lumbar puncture, from which between 1 and 10 mL of the fluid is extracted, depending on the patient [10]. The gold standard for diagnosing bacterial meningitis is CSF cell culture. Gram staining is also often used as a secondary study to confirm this diagnosis; if the stains are negative, a viral source’s likelihoods increased [11]. A relevant sign of viral meningitis is lymphocyte pleocytosis; typically, the white cell count in CSF is in the range of 20–500 cells per milliliter [12]. The complexity of the evaluation to differentiate between BM and VM, along with the small CSF sample volume, has led to the need for a fast method to simplify the analysis. Obtaining the viscosity value in a low volume CSF sample will allow this discrimination to be carried out in an agile and efficient manner.

Quartz Crystal Resonators (QCRs) are devices based on the piezoelectric properties of quartz, used as thickness-shear-mode acoustic wave devices whose applications are based on their resonance frequency changes when the crystal surface is in contact with a small deposited load. Their use as sensors ranges from space applications [13] to chemical-biological applications [14,15], with the Quartz Crystal Microbalance (QCM) being their most common application [16,17,18]. Their use as diagnostic tools has been growing by using biological fluids for disease detection, for example: blood [19,20,21], urine [22], and saliva [23]. An extensive review of studies using QCM for the detection of diseases, such as Influenza, Hepatitis B, Dengue, Malaria, Human Immunodeficiency Virus, and tuberculosis, is presented in Reference [24], with promising initial results. Despite its efficiency and short detection time, this evaluation comprises the crystal electrode’s biofunctionalization to detect the proteins related to the disease, which makes the measurement process complex. On the other hand, there are studies where QCRs have been used to measure different fluid’s viscosity. Measurements with droplets of different industrial oils were performed in Reference [25] to establish the oil’s working state. The use of QCRs to measure battery lead-acid viscosity to determine the state of charge of the battery is shown in [26]. In Reference [27], a method is shown to determine density and viscosity separately by measuring droplets of various fluids. A previous study of viscosity measurements with Hyaluronic Acid (HA) was performed in our laboratory, and the results are shown in Reference [28]. Since QCRs have been used to measure fluids viscosity and need a very small volume of sample to work with, they are good candidates for the new prototype and associated methodology.

In this work, two sensors are used to measure two fluids whose viscosities have different behavior from a rheological point of view: non-Newtonian (SF) and Newtonian (CSF) behavior. The first one, named “ViSQCT”, was developed in-home at Universidad Politécnica de Madrid, and the second, a commercial one from Open QCM-Novaetech S.r.l. company (Naples, Italy). We compare the commercial QCR with ours because they both obtain the frequency shift by different methods, allowing us to get deep insight into our methodology. A conventional viscometer, that needs much more volume of sample than normally clinically available, thus making it useless as a diagnostic device, was used as a reference for calibration purposes. The development phantoms of SF and CSF, that is, fluids that simulate SF and CSF physical properties and whose viscosity can be measured with the sensors is also shown. We finally discuss on the capabitlity of ViSQCT to measure sample’s viscosities. Differentiating between healthy and pathological fluid phantoms can be used to provide a diagnostic method of several pathologies related to the studied biological fluids. However, some differences in the performance between both QCR-based systems, the commercial one and ours, with the reference system and between them, deserve further study and explanation.

## 2. Materials and Methods

### 2.1. Quartz Crystal Resonator

As mentioned above, one of the main applications of quartz crystal resonators is as a microbalance (QCM), whose origin derives from the research conducted by Sauerbrey, which describes the relationship between the crystal resonance frequency shift and the added mass (Equation (1)) [29]. When the mass placed on the crystal surface is replaced by a fluid, the crystal surface movement generates a shear wave that propagates into the deposited fluid; consequently, this wave will suffer a damping effect. The Kanazawa relationship gives the connection between the frequency shift and the fluid’s density-viscosity product in contact with the crystal (Equation (2)) [30]. However, this relationship has been derived theoretically for an infinite quartz plate without interfacial slip and for the case of a perfectly smooth surface; consequently, the use of this equation has not proved to be reliable when measuring the density-viscosity product of Newtonian fluids [31,32].

(1) $$\Delta f=-\frac{2f_{0}^{2}}{A\sqrt{\rho_{q}G_{q}}}\Delta m,$$

(2) $$\Delta f=-\sqrt{n}f_{0}^{3/2}\sqrt{\frac{\rho_{L}\eta_{L}}{\pi \rho_{q}G_{q}}},$$

(3) $$\Delta f=f_{s}-f_{0},$$

where $\rho_{q}=2.648$ g · ${\mathrm{cm}}^{-3}$ and $G_{q}=2.947\times 10^{10}$ N · $m^{-2}$ are the specific density and the shear modulus of quartz, respectively, $f_{0}$ is the fundamental resonance frequency of the quartz, $f_{s}$ is the resonance frequency of the crystal loaded, A is the piezoelectrically active crystal area, Δm is the thin film of mass deposited, $\rho_{L}$ is the fluid’s density, $\eta_{L}$ is the fluid’s viscosity, Δf is the frequency shift, and, finally, *n* is the overtone number; in this work, fundamental frequency of the crystal is used; thus, *n* will be 1.

**Figure 1:**
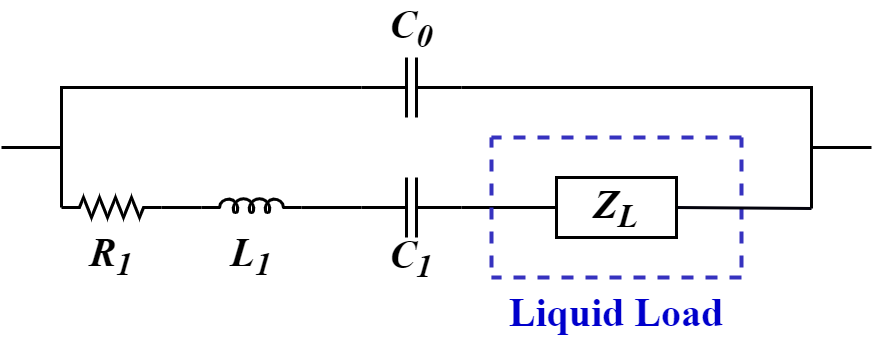
Butterworth-Van Dyke model of Quartz Crystal Resonators (QCR) with liquid load.

A common representation of the crystal is the electrical circuit known as the Butterworth-Van Dyke (BVD) equivalent circuit (Figure 1) derived from Mason equivalent circuit [33]. This model explains the crystal behavior near its resonance frequency and is composed of two arms; the first is the motional arm, and it has three series components: $R_{1}$, $L_{1}$, and $C_{1}$, $R_{1}$ models the dissipation of the oscillation energy from the medium in contact with the crystal, $L_{1}$ is the inertial component of the oscillation and is related to the displaced mass while the crystal is vibrating, $C_{1}$ models the stored energy and is associated with the elasticity of the quartz. The second arm contains a parasitic capacitance in parallel ($C_{0}$); this capacitance is related to the sum of the crystal’s electrodes’ static capacitances. When the crystal is in contact with a liquid load, one element is added to the first branch, $Z_{L}$ is the loading from the liquid sample [34].

In Reference [35], the admittance for the circuit is expressed:

(4) $$Y=\frac{1}{R_{1}+j\omega L_{1}+\frac{1}{j\omega C_{1}}+Z_{L}}+j\omega C_{0},$$

where $Z_{L}$ is the viscosity from the liquid load:

(5) $$Z_{L}=\frac{t^{2}}{4e_{35}^{2}S}\left[\sqrt{\frac{\omega \rho_{L}\eta_{L}}{2}}+j\sqrt{\frac{\omega \rho_{L}\eta_{L}}{2}}\right],$$

where *t* is the thickness of the quartz plate, ω is the angular frequency, $e_{35}^{2}$ is the piezoelectric constant, and *S* is the electrode area. In addition, the conductance is expressed as:

(6) $$G=\frac{R_{1}+R_{e}\left[Z_{L}\right]}{{\left(R_{1}+R_{e}\left[Z_{L}\right]\right)}^{2}-{\left(\omega L_{1}+Im\left[Z_{L}\right]-\frac{1}{\omega C_{1}}\right)}^{2}}.$$

A resonance frequency can be achieved from the equivalent circuit called the series resonance frequency (or series minimum impedance frequency), which occurs at the maximum conductance point. According to the electrical model, when the crystal is in contact with a fluid, the conductance peak is displaced in frequency to a minor frequency and decreasing its magnitude; this maximum conductance can be located by performing a frequency sweep that encloses the fundamental resonance frequency of the crystal.

### 2.2. Measurement Setup

**Figure 2:**
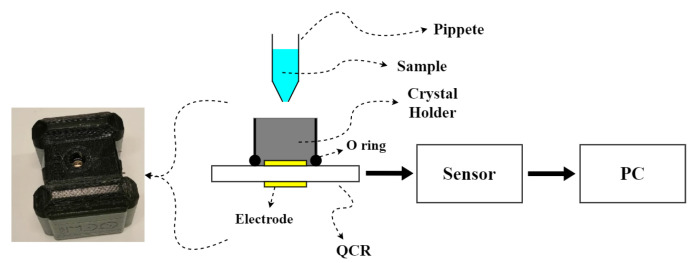
Experimental set up. The sample is poured onto the QCR.

The experimental setup is illustrated in Figure 2; the QCR is placed inside a 3D-printed holder cell where the liquid sample is dropped by pipette and allows the static measurement of the liquid; the volume of sample used is 50 μL since it allows to cover the electrode for the required time fully and to avoid the complete evaporation of the sample. Experiments were performed at room temperature. The QCR is connected to the respective sensor to measure the resonance frequency. The sensors are controlled by software using a computer, where the data is also stored and analyzed. The same measurement protocol was followed with both sensors. First, the resonance frequency of the crystal (without sample) was measured for 5 min, then the sample was deposited in the crystal and the resonance frequency was measured again for another 5 min. The data obtained during the 5 min of each measurement were averaged to obtain the values of $f_{0}$ and $f_{s}$ required to calculate Δf. Once Δf has been obtained, the sample’s viscosity was calculated using Equation (2).

AT-cut quartz crystals with the fundamental resonance frequency of 10 MHz, roughness <1 nm, and gold electrodes were used. The crystals were purchased from Krystaly (Hradec Králové, Czech Republic). Crystal cleaning after experiments was carried out by adding abundant non-ionized water, which was then rinsed with alcohol (isopropanol), and then rinsed again with non-ionized water. Finally, the electrode surface was dried with air.

**Figure 3:**
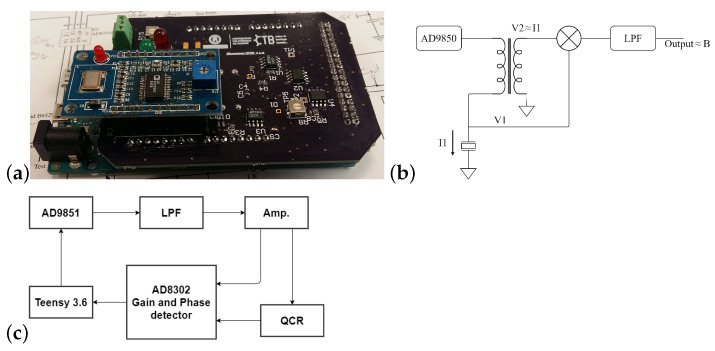
(**a**) ViSQCT sensor. (**b**) ViSQCT sensor simplified diagram of the signals acquisition system. (**c**) Open QCM^®^ signal acquisition method.

As a reference, a rotational viscometer (Alpha series, Fungilab^TM^, Barcelona, Spain) was used. LCP adapter was employed; this allows viscosity measures up to 2 Pa · s using 16 mL of the fluid. The viscosity results obtained with the sensors were compared with those obtained with the rotational viscometer. An earlier version of the ViSQCT sensor is shown in Reference [36]; the current prototype has slight improvement, such as a gain control via digital potentiometers and the exchange of through-hole parts by SMD (surface-mount technology) elements that reduce its size (Figure 3a). Resonance frequency obtention is achieved by exciting the crystal with a frequency sweep near the fundamental resonance frequency and measuring the voltage, current, and susceptance to find the frequency where maximum conductance is located. A simplified diagram of the acquisition signals is observed in Figure 3b.

Open QCM Q-1^®^ (Novaetech S.r.l., Naples, Italy) is the second sensor used, an open-source sensor capable of measuring the resonance frequency, making a frequency sweep, and using a gain-phase detector which compares the exciting signal entering to the crystal with the signal at the crystal output [37]. The device used was adjusted to become compatible with the crystals used in our experiments by adding a pair of clip wires to the device’s output connecting to the crystal. Figure 3c illustrates a block diagram of the acquisition method of the system.

Although both devices can measure the resonance frequency with a frequency sweep, the range covered by this sweep is different. In the ViSQCT sensor, the frequency range is 50 kHz, allowing longer measurement times and a higher resolution when measuring larger Δf (high viscosities). On the other hand, the Open QCM^®^ sensor has a frequency sweep range of 11 kHz, making the measurement capture faster but difficult when measuring very large Δf.

### 2.3. Samples

Distilled water, acetone, isopropyl alcohol, and different dilutions of mixtures of glycerin, sugar, and salt were employed to study the measuring characteristics with simple fluids. The dilutions used were 10, 20, and 30 wt% to increase the density-viscosity of the fluid. An analytical balance (Discovery dv215, Ohaus^TM^, Nänikon, Switzerland) was used to weigh the solute of the samples and to obtain the density by weighing 1 mL of sample volume.

#### 2.3.1. Artificial Synovial Fluid

SF is a mixture of plasma and Hyaluronic Acid (HA), whose concentration determines the fluid’s viscoelastic properties. Arthritic diseases are associated with the reduction of HA [2,4,9,38]. For this reason, HA dilutions were made to generate artificial SF (aSF) and emulate physical properties, such as viscosity. In healthy SF, the concentration is around 3.5 mg/mL, while, in Osteoarthritis (OA), the HA concentration decrease to 1.3 mg/mL, and, in Rheumatoid Arthritis (RA), to approximately 0.84 mg/mL [7].

Six dilutions were made, one for healthy fluid, two for abnormal fluid using the concentrations mentioned above. The other three dilutions were created to test the sensor’s detection capabilities better. The mixtures were made with distilled water and weighing the necessary amount of HA with an analytical balance. After adding HA (Mw = 1.5 MDa; Acros Organics Inc., Geel, Belgium) into the water, gentle stirring was required. Finally, the sample is stored in the refrigerator for a few hours to eliminate bubbles formed when mixing. Table 1 shows the compositions of the SF phantom produced.

#### 2.3.2. Artificial Cerebrospinal Fluid

CSF is a complex fluid mainly composed of Na, Cl, and ${\mathrm{HCO}}_{3}$ with lesser amounts of K, Mg, Ca [5,39]. Some artificial Cerebrospinal Fluid (aCSF) are reported in References [40,41], where NaCl and ${\mathrm{NaHCO}}_{3}$ are the elements with higher concentrations. According to this, healthy artificial CSF was developed with the concentrations listed in Table 2. To increase viscosity in aCSF and simulate abnormal fluid, albumin was used since it is the protein with a higher concentration in pathological CSF [42,43]. Two extra samples were measured to have more information on the detection abilities of the sensors.

## 3. Results

Measurements were made following the procedure mentioned in previous sections. The results were obtained by averaging five independent measures of each fluid.

### 3.1. Pure Fluids: Water, Alcohol, and Acetone

**Figure 4:**
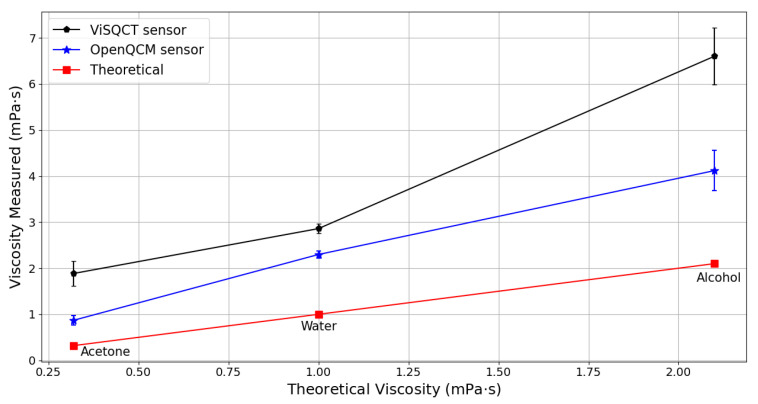
Comparison of measured and theoretical viscosities of: Water, Acetone, and Alcohol.

In Table 3, the results for Water, Alcohol, and acetone are presented. Measured Δf and the theoretical values of density and viscosity of the fluids are shown. The three liquids have different viscosities, with acetone being the least viscous and isopropyl alcohol the most. It can be noted that the frequency difference increases along with the density-viscosity of the fluid. Knowing the sample’s density value, the viscosity can be expressed from the measured Δf. The difference between the theoretical viscosity value and the viscosity obtained by each sensor is significant, where it is superior in case of the ViSQCT sensor. For example, the viscosity of water is around 1 mPa · s (20 °C), and the results show a value of 2.86 mPa · s for the ViSQCT sensor and 2.29 mPa · s for the Open QCM^®^ sensor. These differences are illustrated in Figure 4.

### 3.2. Test Dilutions: Glycerin, Sugar, and Salt Dilutions

**Figure 5:**
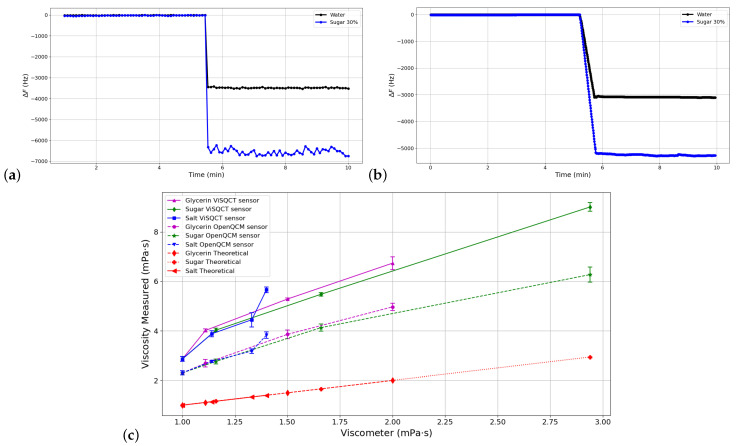
Resonance frequency measurement process for water and sugar 30% cases using: (**a**) ViSQCT sensor. (**b**) Open QCM^®^ sensor. (**c**) Comparison of measured viscosities for different Glycerin, Sugar, and Salt dilutions in water using the two sensors and the rotational viscometer.

According to the methodology described above, measurements were made using glycerin, sugar, salt in water dilutions of 10, 20, and 30 wt% to increase viscosity. Figure 5a,b shows the Δf measurement process (normalized) using water (black line) and sugar 30% (blue line) samples with both sensors. In both cases, the frequency drop is observed when the sample is added. In addition, stability is noted when measuring the resonance frequency, where the Open QCM sensor has a more stable curve when measuring sugar 30% (the sample with the highest viscosity of the set).

The dilution’s viscosity results are shown in Table 4 and illustrated in Figure 5c. The dashed line represents the data obtained with the Open QCM^®^ sensor. The continuous line represents the ViSQCT sensor data; the value measured with water is also shown in the figure. As expected, the viscosity and Δf proportionally increase in each fluid. There is a similar trend in Δf increase between both sensors with an offset of approximately 1.5 mPa · s. As in the previous section results, the differences between the viscosities obtained with the viscometer measurements are large, but it is possible to distinguish the resulting frequency change for each sample.

### 3.3. aSF Results

**Figure 6:**
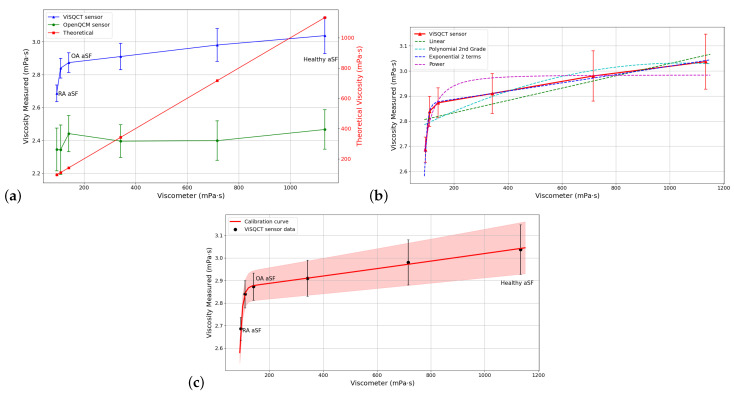
(**a**) Comparison of measured viscosities for aSF using the two sensors and the rotational viscometer. (**b**) Adjustment with different functions for aSF. (**c**) Calibration curve for aSF.

The results of the measurements using aSF are exposed in Table 5; it can be observed that, as the HA concentration increases, the viscosity also increases. For healthy aSF, the resulting viscosity with ViSQCT sensor is near 3 mPa · s. For RA aSF (lowest viscous sample), the viscosity is 2.68 mPa · s. In Figure 6a, the results are illustrated; the blue line represents the data from the ViSQCT sensor and the green line for the Open QCM^®^ sensor. The theoretical curve (red line), which is the data measured with the viscometer, is also shown on a different scale. For the suggested methodology, the Open QCM^®^ sensor presents difficulties in measuring this type of fluids having an almost flat response; it is hardly possible to notice an increase in viscosity between the low and high HA concentration samples. The ViSQCT sensor curve shows that discrimination between healthy aSF and RA aSF is difficult due to a slight overlap between the maximum threshold of RA aSF and the minimum threshold of healthy aSF; however, it allows differentiating healthy aSF from RA aSF.

Due to the difference between the viscosity values obtained with the sensor and the viscometer, it is necessary to find an adjustment curve, that allows the sensor’s calibration. Table 6 shows the comparison between different curves for fitting the viscosity results and the RMSE (root-mean-square error) of each fit. The curve with the lowest error is the exponential curve having an RMSE of 0.0073; in Figure 6b, this is clearly noted; hence, this function will be used as the calibration function for the aSF.

The fitting is performed for the data obtained with the ViSQCT sensor. However, the procedure can be repeated to fit more results.

Figure 6c illustrates the sensor viscosity points together with the selected calibration curve. The effectiveness of the adjustment can be observed as all the measured points are seen on the curve. After the calibration process, the final viscosity obtained is close to that measured with the rotational viscometer, as shown in Table 7.

### 3.4. aCSF Results

**Figure 7:**
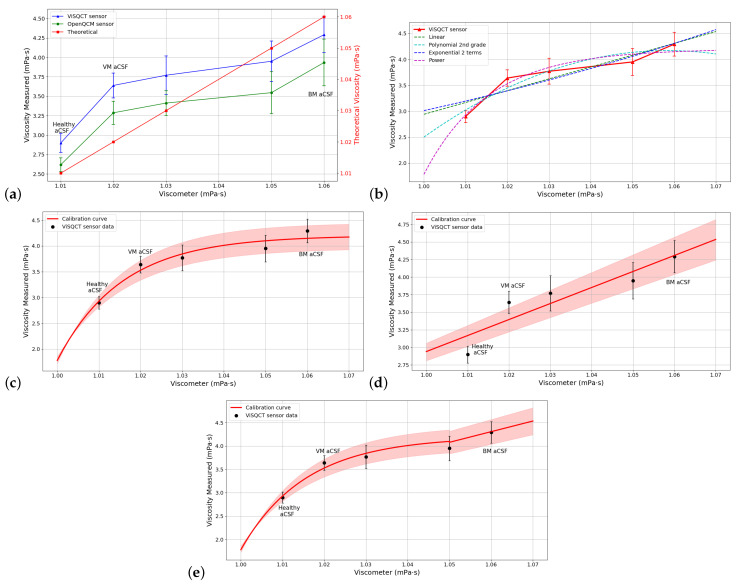
(**a**) Comparison of measured viscosities for aCSF using the two sensors and the rotational viscometer. (**b**) Adjustment with different functions for aCSF. (**c**) Power function adjustment for aCSF. (**d**) Linear adjustment for aCSF. (**e**) Calibration curve using a function defined by parts for aCSF.

The results from the measurements using aCSF are presented in Table 8. Results with both sensors were similar; for example, for the healthy aCSF (lowest viscosity), the viscosity measured with the ViSQCT sensor is 2.898 mPa · s, and the one obtained with the Open QCM^®^ sensor is 2.618 mPa · s, this is almost 0.3 mPa · s of difference, this difference increase to 0.35 mPa · s for the most vicious case, BM aCSF. In Figure 7a, the comparison between viscosities obtained with the viscometer and with the sensors is shown. Again, the blue line represents the ViSQCT sensor data and the green line for the Open QCM^®^ sensor. The theoretical curve (red line), which is the data measured with the viscometer, is also shown on a different scale. The two sensors could detect viscosity changes between healthy aCSF fluid, VM aCSF fluid, and BM aCSF fluid. The trend is similar between both sensors, with the Open QCM^®^ sensor being the closest to the ideal values.

Table 9 shows the comparison between different curves for fitting the viscosity results for aCSF; also, the RMSE of the fit is noted. In Figure 7b, all the curves are shown. The curve with the lowest error is the Power function curve having an RMSE of 0.176, which is marked in the graphic.

The Power function is plotted with the measured points in Figure 7c, the points are almost in the function line, but for the higher viscosity data, this curve will reach a limit; thus, the adjustment for high viscosities is not efficient, for a second-degree polynomial function this effect will remain. In Figure 7d, the adjustment using a linear function is illustrated; for this case, the lowest viscosity values present a high error, only having the BM aCSF near the adjustment line. A function defined by parts is proposed for aCSF sample having the power function in the range (1, 1.05) and the linear function for the second range (1.05, 1.7); using this function, the RMSE is reduced to 0.0899, and the differentiation between the healthy aCSF, VM aCSF, and VM aCSF is feasible. The calibration curve is observed in Figure 7e. After the calibration process, the final viscosity obtained is close to that measured with the rotational viscometer, as shown in Table 10.

## 4. Discussion

The results obtained show that, with the proposed method, there is an offset phenomenon concerning the measurements made with the rotational viscometer. On the other hand, [27] shows a different method for measure density and viscosity separately using volumes from 2 to 10 microliters (by drops); the results present a slight deviation concerning the theoretical values, the authors mention that the cleanliness of the crystal, the working state of the QCR circuit, and the temperature of the environment as the main sources of the error. In Reference [31], Johannsmann mentions that certain effects interfere with the measuring of viscosity originated for nanoscopic air bubbles, roughness, slip, and compressional waves. In addition, mentions the importance of differentiating high-frequency viscosity from the viscosity in steady shear. For this reason, it is necessary to perform a calibration of the device for each sample and, thus, have the way to convert the measured data to real data measured with a rotational viscometer. Despite this, the application of this new form of measurement yields results that show its optimal ability to detect viscosity changes in fluids aiming to detect pathologies associated with these viscosity changes.

Both sensors have different approaches, ViSQCT was designed to measure the Δf associated to a fluid’s viscosity by performing a static measurement, while the Open QCM^®^ sensor is usually used more as a microbalance by flowing the sample with a microfluidic system. The two sensors performed adequately in measuring the Δf caused by the density-viscosity of the measured fluid and with similar uncertainties. The Open QCM^®^ sensor obtained a slightly lower offset than the ViSQCT sensor. However, for the case of aSF, it presented difficulties to measure this fluid adequately. The main reason is that the increment of the half-bandwidth caused by these fluids is wide, and the frequency sweep range was not able to detect efficiently the resonance frequency creating saturation when measuring. With this in mind, the ViSQCT sensor is more versatile as it can measure different types of fluids and larger Δf.

In the particular case of aSF, the Δf obtained corresponds to viscosity values lower than the measured with the rotational viscometer; since HA concentrations are a non-Newtonian fluid with pseudoplastic behavior, this means that at the higher shear rate, the viscosity will decrease. The rotational viscometer measures at 0.3 rpm; thus, it will measure a higher viscosity with HA since the fluid is handled with a low share rate. On the other hand, the crystals are vibrating near 10 MHz which can be considered a high shear rate; thus, the viscosity will be minor. Comparison between low share rate viscosities with high share rate viscosities is beyond the scope of this work; however, the differentiation between healthy aSF from RA aSF is possible. Differentiation of OA aSF is difficult due to the uncertainties of the data measured. The viscosities of aSF obtained with the sensors are similar to those obtained in a previous study [28].

In the case of the aCSF samples, both sensor’s performance in discriminating healthy aCSF, VM aCSF, and BM aCSF were possible. Comparing the viscosities measured with the sensors and with the rotational viscometer, it can be seen that the sensors have a greater measurement sensitivity than the rotational viscometer.

The ViSQCT sensor proved to be useful for measuring viscosity changes; however, it must be improved to reduce the observed errors. The study of the half-band half-width value in the viscosity calculation could contribute overcome those errors. In Reference [3], viscosity measurements for CSF were done at 37 °C, obtaining viscosities less than 1 mPa · s and with a minimal variation between healthy CSF (0.72 mPa · s) and CSF with bacterial meningitis (0.87 mPa · s). In this work, measurements were performed at room temperature for technical reasons without having difficulties in the differentiation of each fluid.

The size (portability) and cost are the main advantages of the proposed prototype since, in experiments where QCRs are used to measure viscosity [25,26,27], large and expensive equipment, such as network analyzers or universal counters, are usually used.

## 5. Conclusions

This paper shows a method to measure human fluid’s viscosity (SF and CSF) using a small sample volume (around 50 μL). From this data, a medical diagnosis could be made; thus, further studies on real biological fluids should be carried out to validate the sensor’s use as a diagnostic tool. The ViSQCT sensor, at least when being applied to phantoms fluids, can distinguish between healthy aSF and RA aSF, but OA SF detection is still difficult. As aSF is a non-Newtonian fluid, the viscosity results obtained with the sensor had a smaller increase than those obtained with the reference viscometer. The results obtained with aCSF show the possibility of detecting the small viscosity changes between the healthy aCSF, VM aSF, and BM aSF. Calibration curves were suggested for each fluid to adjust the sensor data with the rotational viscometer information. Due to the portability, low cost, and simplicity of the proposed method, the prototype deserves further development aiming to become a device able to assist physicians in making a prompt diagnosis using a small volume sample for the cases shown in the present work.

## Figures and Tables

**Table 1:** Artificial Synovial Fluid (aSF) compositions (error = 0.01).

Fluid	H. A. Concentration (mg/mL)
Healthy aSF	3.5
aSF3	3.0
aSF2	2.0
OA aSF	1.3
aSF1	1.0
RA aSF	0.84

**Table 2:** Artificial Cerebrospinal Fluid (aCSF) compositions (error = 0.01).

Fluid	NaCl Concentration (mg/mL)	${\mathrm{NaHCO}}_{3}$ Concentration (mg/mL)	Albumin Concentration (mg/mL)
Healthy aCSF	7.25	2.18	0.0
VM aCSF	7.25	2.18	0.5
aCSF1	7.25	2.18	1.0
aCSF2	7.25	2.18	2.0
BM aCSF	7.25	2.18	3.0

**Table 3:** Water, Alcohol, and Acetone results.

Fluid	Density (Theory) (mg/mL)	Viscosity (Theory) (mPa · s)	ViSQCT			Open QCM^®^		
			|Δf| (Hz)	Viscosity Obtained (mPa · s)	Difference Theory vs. ViSQCT (%)	|Δf| (Hz)	Viscosity Obtained (mPa · s)	Difference Theory vs. Open QCM (%)
Water	1000	1.0	3416 ± 62	2.86 ± 0.10	186	3062 ± 54	2.298 ± 0.08	129
Alcohol Isopropanol	786	2.1	4601 ± 218	6.602 ± 0.62	214	3634 ± 196	4.119 ± 0.44	96
Acetone	791	0.32	2467 ± 179	1.886 ± 0.27	489	1676 ± 95	0.87 ± 0.10	171

**Table 4:** Glycerin, Sugar, and Salt concentrations parameters and results with both sensors.

Fluid	Density (mg/mL)	Viscometer (mPa · s)	ViSQCT			Open QCM^®^		
			|Δf| (Hz)	Viscosity Obtained (mPa · s)	Difference vs. Viscometer (%)	|Δf| (Hz)	Viscosity Obtained (mPa · s)	Difference vs. Viscometer (%)
Glycerin 10%	1034	1.11	4115 ± 34	4.015 ± 0.06	261	3367 ± 95	2.687 ± 0.15	142
Glycerin 20%	1064	1.50	4789 ± 20	5.284 ± 0.04	252	4091 ± 96	3.856 ± 0.18	157
Glycerin 30%	1091	2.00	5475 ± 106	6.735 ± 0.26	236	4702 ± 67	4.968 ± 0.14	148
Sugar 10%	1045	1.16	4144 ± 46	4.028 ± 0.08	247	3432 ± 64	2.763 ± 0.10	138
Sugar 20%	1096	1.66	4949 ± 34	5.478 ± 0.07	230	4296 ± 74	4.128 ± 0.14	148
Sugar 30%	1139	2.94	6469 ± 65	9.007 ± 0.18	206	5398 ± 135	6.271 ± 0.30	113
Salt 10%	1088	1.14	4150 ± 64	3.880 ± 0.12	240	3502 ± 25	2.763 ± 0.04	142
Salt 20%	1145	1.33	4557 ± 149	4.446 ± 0.29	234	3855 ± 56	3.182 ± 0.09	139
Salt 30%	1194	1.40	5249 ± 52	5.657 ± 0.11	304	4317 ± 76	3.826 ± 0.13	173

**Table 5:** aSF parameters and results with both sensors.

H. A. Concentration (mg/mL)	Density (mg/mL)	Viscosity (mPa · s)	ViSQCT			Open QCM^®^		
			|Δf| (Hz)	Viscosity Obtained (mPa · s)	Difference vs. Viscometer (%)	|Δf| (Hz)	Viscosity Obtained (mPa · s)	Difference vs. Viscometer (%)
0.84 (RA aSF)	1007	94	3322 ± 31	2.686 ± 0.05	97	3103 ± 88	2.344 ± 0.13	97
1.0	1008	109	3417 ± 36	2.839 ± 0.06	97	3104 ± 99	2.343 ± 0.15	98
1.3 (OA aSF)	1009	141	3439 ± 34	2.873 ± 0.06	98	3170 ± 75	2.441 ± 0.11	98
2.0	1013	342	3468 ± 48	2.91 ± 0.08	99	3146 ± 70	2.395 ± 0.10	99
3.0	1022	716	3525 ± 54	2.98 ± 0.10	99	3162 ± 78	2.398 ± 0.12	99
3.5 (Healthy aSF)	1028	1133	3569 ± 69	3.037 ± 0.11	99	3216 ± 77	2.466 ± 0.12	99

**Table 6:** Comparison between fitting curves for aSF results.

Fitting Curve Type	Function	Parameters	RMSE
Linear	*f*(*x*) = *ax + b*	*a* = $2.458\times 10^{-4}$ *b* = 2.784	0.0728
Polynomial 2nd grade	*f*(*x*) = $ax^{2}$ + *bx + c*	*a* = $2.694\times 10^{-7}$ *b* = $5.634\times 10^{-4}$ *c* = 2.737	0.0747
Exponential (2 terms)	$f\left(x\right)=ae^{bx}+ce^{dx}$	*a* = 2.855 *b* = $5.587\times 10^{-5}$ *c* = −7414 *d* = −0.1128	0.0073
Power	$f\left(x\right)=ax^{b}+c$	*a* = $1.762\times 10^{4}$ *b* = −2.438 *c* = 2.984	0.0563

**Table 7:** Resulting viscosity after calibration for aSF.

H. A. Concentration (mg/mL)	Viscosity Obtained (mPa · s)	Viscosity Calibrated (mPa · s)	Viscosity with Viscometer (mPa · s)	Error (%)
0.84 (RA aSF)	2.686	94.007	94	0.007
1.0	2.839	109.13	109	0.119
1.3 (OA aSF)	2.873	130.70	141	7.305
2.0	2.91	341.50	342	0.146
3.0	2.98	767.00	716	7.123
3.5 (Healthy aSF)	3.037	1106.00	1133	2.383

**Table 8:** aCSF parameters and results with both sensors.

H. A. Concentration (mg/mL)	Density (mg/mL)	Viscosity (mPa · s)	ViSQCT			Open QCM^®^		
			|Δf| (Hz)	Viscosity Obtained (mPa · s)	Difference vs. Viscometer (%)	|Δf| (Hz)	Viscosity Obtained (mPa · s)	Difference vs. Viscometer (%)
0.0 (aCSF)	1008	1.01	3452 ± 75	2.898 ± 0.12	187	3281 ± 61	2.618 ± 0.09	159
0.5 (VM aCSF)	1010	1.02	3872 ± 86	3.639 ± 0.16	256	3680 ± 88	3.287 ± 0.15	222
1.0	1012	1.03	3945 ± 134	3.77 ± 0.25	266	3753 ± 91	3.412 ± 0.16	231
2.0	1013	1.05	4040 ± 136	3.95 ± 0.26	276	3828 ± 145	3.546 ± 0.27	237
3.0 (BM aCSF)	1017	1.06	4220 ± 118	4.292 ± 0.23	285	4040 ± 182	3.934 ± 0.30	271

**Table 9:** Comparison between fitting curves for aCSF results.

Fitting Curve Type	Function	Parameters	RMSE
Linear	*f*(*x*) = *ax + b*	*a* = 22.8 *b* = −19.86	0.2382
Polynomial 2nd grade	$f\left(x\right)=ax^{2}+bx+c$	*a* = −489.9 *b* = 1037 *c* = −544.6	0.2253
Exponential (2 terms)	$f\left(x\right)=ae^{bx}+ce^{dx}$	*a* = 0.00768 *b* = 5.971 *c* = 0 *d* = −216.9	0.4358
Power	$f\left(x\right)=ax^{b}+c$	*a* = −2.422 *b* = −64.6 *c* = 4.203	0.1760

**Table 10:** Resulting viscosity after calibration for aCSF.

Albumin (mg/mL)	Viscosity Obtained (mPa · s)	Viscosity Calibrated (mPa · s)	Viscosity with Viscometer (mPa · s)	Error (%)
0.0 (aCSF)	2.898	1.009	1.01	0.099
0.5 (VM aCSF)	3.639	1.022	1.02	0.196
1.0	3.77	1.027	1.03	0.291
2.0	3.95	1.035	1.05	1.428
3.0 (BM aCSF)	4.292	1.059	1.06	0.094

## Data Availability

The data presented in this study are available on request from the corresponding author.
